# Exploration of underlap induced high-k spacer with gate stack on strain channel cylindrical nanowire FET for enriched performance

**DOI:** 10.1038/s41598-024-53487-1

**Published:** 2024-02-05

**Authors:** Rasmita Barik, Rudra Sankar Dhar, Mousa I. Hussein

**Affiliations:** 1 Department of ECE, NIT Mizoram, Chaltlang, Aizawl, Mizoram India; 2 Department of Electrical Engineering, United Arab Emirates University, Al Ain, United Arab Emirates

**Keywords:** Engineering, Electrical and electronic engineering

## Abstract

This research explores a comprehensive examination of gate underlap incorporated strained channel Cylindrical Gate All Around Nanowire FET having enriched performances above the requirement of the 2 nm technology node of IRDS 2025. The device installs a combination of strain engineering based quantum well barrier system in the channel region with high-k spacers sandwiching the device underlaps and stack high-k gate-oxide. The underlaps are prone to parasitic resistance and various short channel effects (SCEs) hence, are sandwiched by HfO_2_ based high-k. This SCE degradations and a strong electric field in the drain-channel region is rendered controlling the leakages. The strain based Nanosystem engineering is incorporated with Type-II heterostructure band alignment inducing quantum well barrier mechanism in the ultra-thin cylindrical channel region creating an electrostatic charge centroid leading to energy band bending and splitting among the two-fold and four-fold valleys of the strained Silicon layer. This provides stupendous electron mobility instigating high current density and electron velocity in the channel. Thereby, the device is susceptible to on-current enhancement via ballistic transport of carriers and carrier confinement via succumbing of quantum charge carriers. The device transconductance, I_on_, I_off_, I_on_/I_off_ ratio are measured and the output performance (I_D_-V_DS_) characteristics is determined providing emphatic enrichments in contrast to the existing gate all-around FETs as well as the 2 nm technology node data of IRDS 2025. Hence, the strained channel Nanowire FET device developed here is presented here as the device of the future for various digital applications, RF applications and faster switching speed.

## Introduction

Today's world is marked by a consistent trend of device structure modernization through material modifications, which leads to on-going transistor scaling. Short channel effects (SCEs) are caused by device downscaling^1^ and the SCE optimization beyond 20 nm technology node is a challenging task. The SCEs obligate a significant impact on electrical properties at the nanoscale, such as mobility deterioration, changes in threshold voltage, and a rise in off-current leakages^2^ leading to generation of hot carriers at nano regime. Furthermore, these hot carriers produce trap charges at the MOSFET structure's Si-SiO_2_ interface, which significantly boosts SCEs and causes subsequent degradations in device performances. Many solutions are being researched to improve device performance and reduce variability, including the invention of nanowire FET (NW FET) ^3^, introduction of high-k gate stack^4^, and substantial channel doping profile^5^, multi-gate structured FETs^6^, and electron mobility augmentation employing strain technology^7^. Many of the recent investigations on strain engineering have also revealed mobility amplification techniques by reducing V_th_^8,9^. Strain in short-channel devices mitigates the current degradation due to parasitic resistance and dipping of voltage by increasing the effective carrier velocity in the device channel. Because of these notable improvements in the strained silicon channel, which allow for higher drive currents without gate oxide scaling, the industry has quickly embraced the transport characteristics of the strained silicon technology. Global strain and local strain approaches are classified as producing biaxial and uniaxial strain, respectively^10^. Strain application has two major effects in nano FETs that are: (i) shifting of the band energy level, (ii) degeneracy level splitting; occurring in the device electronic states. The minima of the conduction band in the four fold valley is detected to have higher energy than the two-fold valley because of the strain in the channel^1^, which induces increased electron occupancy in the two fold valleys and energy band splitting. As a result of this scenario, there is an increase in electron mobility, which inhibits the intermittent electron transition from the lower valley to higher valley and reduces scattering effects in the ultrathin channel^11^. DG FETs, Fin FETs, and GAA (Gate All Around) FETs are few examples of multi-gate devices that have gradually evolved over the past decades with a rise in electric controllability through increasing the number of gate electrodes in the device^12^. Whereas the gate in a Fin FET covers the channel region on three sides, the Nanosheets are surrounded by the gate, classifying the device as a GAA device. This architecture leads to improved electrostatic control of the transistor, faster transistor switching speeds, and acceptable driving currents in a smaller footprint. Hence, GAA FET is destined as one of the promising new technologies, which has gates on all sides. Because it provides higher device performance at lower dimensions, GAA FET technology is expected to be the successor to Fin FETs. GAA transistors are built using nanowire and Nanosheet structures. Depending on the implementation, the GAA FET structures may be parallel or perpendicular to the substrate. Gate-all-around devices are majorly classified into two types: rectangular (RGAA) and cylindrical (CGAA). The channel region of the RGAA device is rectangular in shape, whereas the channel of the CGAA device is cylindrical. CGAA technology outperforms RGAA technology in terms of gate electric controllability, lessens corner impacts^13^, and negates variety SCEs. Despite these aspects cylindrical GAA devices outperforms^14^, drawbacks such as high leakage current and subthreshold swing render them inappropriate for low power and higher speed switching needs.

Conventional FETs are scaled down to nano-regime and the SiO_2_ layer used as the gate dielectric becomes thinner leading to increase in gate leakage current and power dissipation. To mitigate these effects, high-k dielectric is used as an alternative material in existing Fin FET technology^15^. The threshold voltage offset generated by dense trap charge and the poly depletion impact, however limits this even further^16^. Growing directly a high-k material on the Si channel region infuses interface scattering effect, hence gate stack high-k is indebted . The gate stack is developed applying a thin high-k layer atop of the silicon dioxide layer in order to get the desired enhanced characteristic while avoiding these constraints. This is achieveable due to the use of high-k considering equivalent oxide thickness (EOT). Considering this aspect many research with high-k gate stack for DGFET^17^, high-k gate stack Fin FET^18^, high-k gate stack CGAA FET^19^, NW GAA MOSFET^20^, gate stacked GAA TFET^21^, and high-k gate stack Junction less GAA^22,23^ are conducted demonstrating reduction in off-current and increase in the switching speed of the devices. Thus GAAs with high-k dielectric materials though not established are being developed in order to lower the channel resistance, enhance switching speed, and optimize SCEs^24^ to be at par with the IRDS 2022^25^. Multiple Nanosheet channels are frequently layered vertically to increase the effective width of the transistor and its driving current. Multi-Bridge Channel FETs (MBCFETs) are Samsung's version of Nanosheet transistor technology^26^, and they are expected to go into production at for 3 nm node. RibbonFET^27^ is the term given to Intel's version of the similar technology. Because designers can adjust the width of the flexible ribbon-based channel, RibbonFET technology can be used in a wide range of switching, amplification, and driving applications. The rising desire for scaling down to 5 nm nodes challenges the current FET technology. RibbonFET overcomes this challenge by using a single stack of nanoribbons as its channel, substantially reducing its footprint for designs with limited area on the wafer. But, these devices for 3 nm and 5 nm nodes are yet to settle in the semiconductor market and is under research.

Stack initiation high-k in FinFETs is successfully achieved, however with the IRDS 2025 proposal under IRDS 2022^25^ redifines the need for smaller and quicker devices, hence a requirement of GAA with stack high-k stands out. In addition, there are many studies on the employment of strained channel DG FETs^28^ and FinFETs^29^, which demonstrate that the strained channel enhances on-current while the stack high-k gate increases switching speed, minimizes off-current, and weakens the subthreshold swing (SS). Furthermore, the settlement of a three-layered strained NW channel GAA system on a 10 nm gate length is observed to be quite spectacular in terms of ON current enrichment^30^. But SCEs, on the other hand leading to off-current leakages and subthreshold swing are present and sometimes higher in these devices so as the desired performances are not achieved. The channel area of the 10 nm strained channel NW GAA FET comprises of ultra-thin tri-layer strained layers that installs ballistic transport of carriers at nano-dimension device due to quantum carrier confinement in the channel region^31,32^, resulting in overall improved device performances. To compete with the IRDS 2025^25^ anticipated 2 nm technology node device, a strained channel NW FET with stack high-k gate at 8 nm channel length is first time developed here. This paper investigates on the performance of gate underlap inducted with the strained channel in NW FETs having stack high-k gate. The effects of gate underlap, strained channel, increasing source and drain radius, and high-k spacers are taken into account while developing the novel NW FETs here, and the performance parameter analysis, including on-and-off current, current ratio, and subthreshold swing, are carried out and compared to the existing CGAA FETs and IRDS requirements to estimate improved performances. As a result, the novel construction that is optimized here for betterment includes a gate underlap with the strained channel system and stack high-K gate in the NW FET, which improves performance by allowing for faster switching due to a higher I_on_/I_off_ ratio, abridged leakage current, and lower SS to be above the 2 nm technology node requirements of IRDS 2025.

## Device theory and arrangement

**Figure 1:**
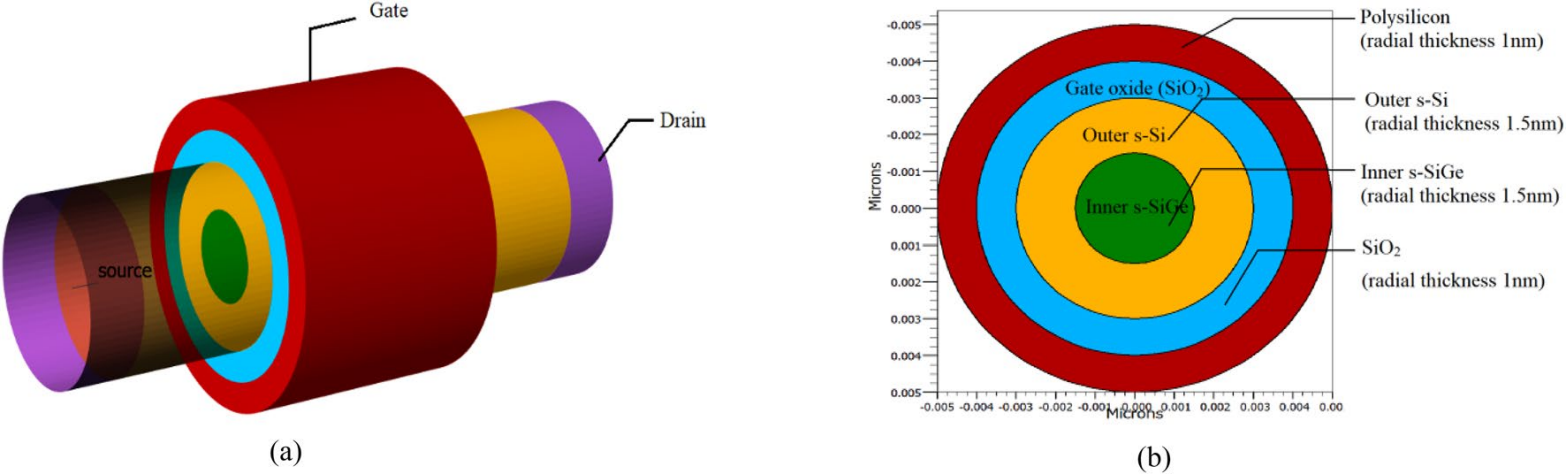
**(a)** The three-demensional schematic of the 8 nm strained channel NW FET and (**b**) cross-sectional of the channel layers of device B.

**Figure 2:**
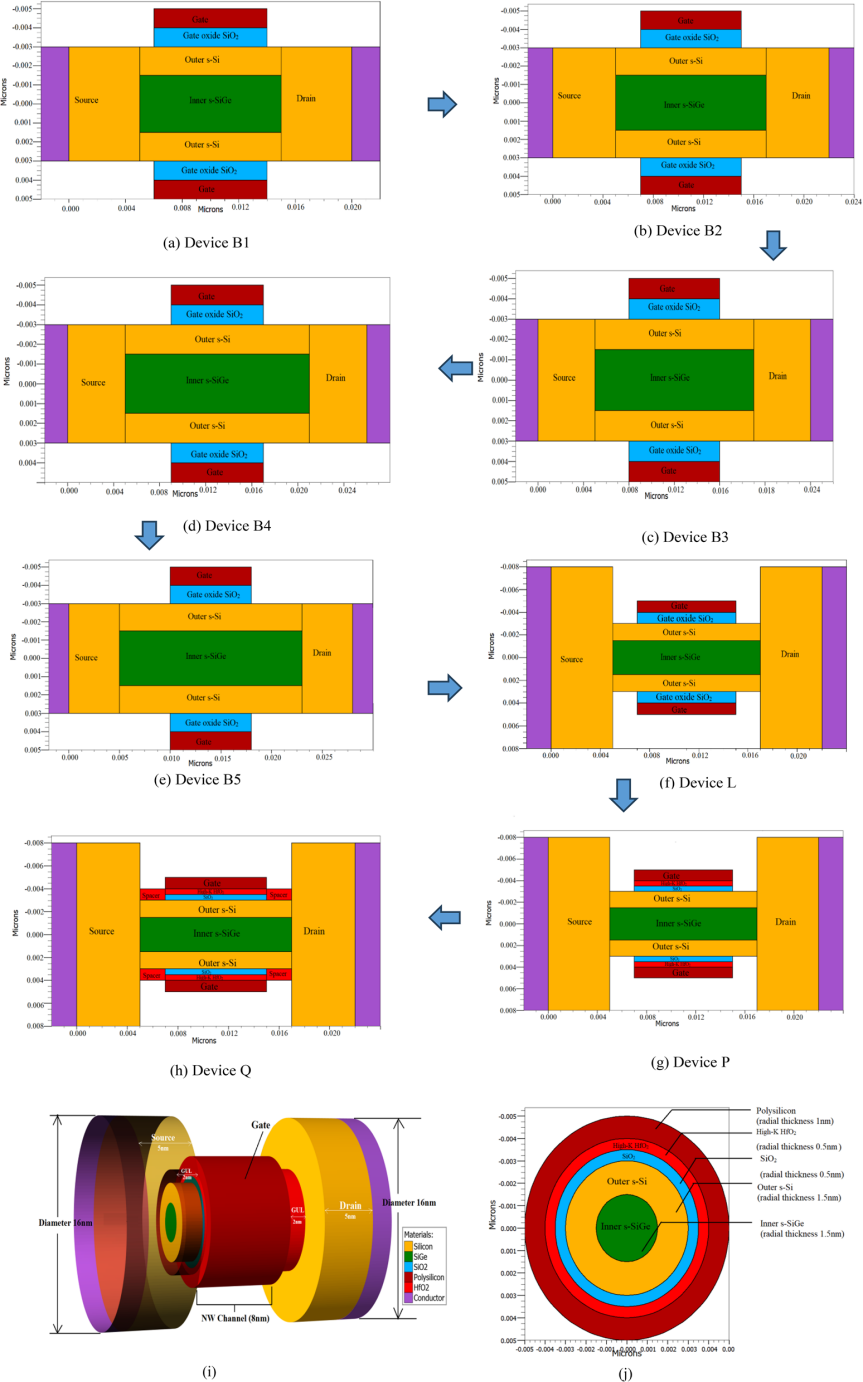
(**a**) Cut-sectional view of device B1, (**b**) cut-sectional view of device B2, (**c**) cut-sectional view of device B3, (**d**) cut-sectional view of device B4, (**e**) cut-sectional view of device B5, (**f**) cut-sectional view of device L, (**g**) cut-sectional view of device P and (**h**) cut-sectional view of device Q, (**i**) the three-demensional schematic of device Q and (**j**) cross-sectional view with channel layers of device Q.

**Figure 3:**
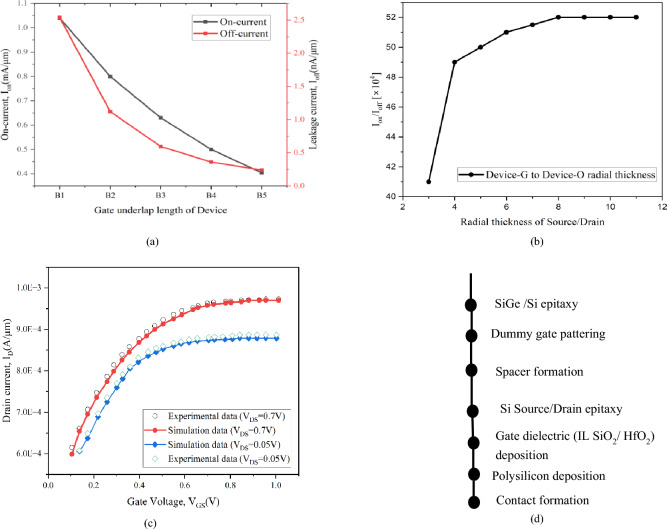
(**a**) Graphical representation of leakage (Off) current and On-current for the devices B1, B2, B3, B4 and B5, (**b**) graphical illustration of the increase in I_on_/I_off_ ratio with change in the source/drain radial thicknesses. (**c**) Electrical parameter fitting for calibration to the experimental data and the physical parameters of the device U^34^ with I_D_–V_GS_ logarithmic graph where V_DS_ represents drain voltage, (**d**) fabrication process flow of NW CGAA FET.

The schematic of the 8 nm gate length strained channel NW FET in three-dimensional is created here for the first time and is shown in Fig. 1a. Here the strained channel consists of strained silicon (st-Si) encapsulating st-SiGe. The NW FET with variations based on channel layer thicknesses (st-Si/st-SiGe) is labelled as Device A through Device F as in Table 1. The radial thickness of the strain Silicon layer (1, 1.5, 2 nm) and the s-SiGe layer (1, 1.5, 2 nm) are considered and varied to make devices A, B, C, D, E, and F, respectively; this is to regulate scattering in the ultra-thin channel, as Leys et al.^33^ claimed for effective straining in nano regime devices the silicon layer thickness can be kept between 1 and 2 nm. With 1 nm being very thin and 2 nm may not being able to enforce strain to the needed extent as observed in Table 2, Device B acquired greater I_on_/I_off_ ratio than Devices A, C, D, E, and F due to its 1.5 nm st-Si thickness and 1.5 nm st-SiGe radial thickness. Consequently, the 1.5 nm thickness of the channel's st-Si and st-SiGe layers are taken into account for continued development of the device as depicted in Fig. 1b. Devices B1, B2, B3, B4, and B5 are then built by ranging the underlap length from 1 to 5 nm from gate to the source and drain side while taking 3 nm source and drain radial width but maintaining the width of the st-Si and st-SiGe layers at 1.5 nm, as illustrated from the sectorial view in Fig. 2a–e. It is observed that by increasing the gate underlap length the decrease in Off current is noteworthy as shown in Fig. 3a. Figure 3a depicts the graphical presentation of on-current and off-current for the 8 nm CGAA FET by varying the underlap length from 1 to 5 nm (Device B1 to Device B5). The figure shows that both on-current and off-current decreases with increase in the underlap length due to increase in parasitic series resistance. The off-current decreases exponentially for Device B1 to Device B5 subdivisions due to lessened tunnelling of carriers from source to channel region as detected in Fig. 3a while also the decrease in on-current is observed with increase in the underlap length due to increased parasitic series resistance. Based on this deliberation as acquired from Fig. 3a of the device performance the off-current variation of the gate underlap NW CGAA FET is calculated and formulated as the novel Exponential Decay Relation given by:

1 $${I}_{off}={k}_{1}+{exp}^{\frac{{l}_{gu}}{{k}_{3}}}+{k}_{2}$$

where I_off_ is the off-current, l_gu_ is the underlap length and K_1_, K_2_ and K_3_ are constants of the exponential decay in off-current for increase in underlap lengths, which stands similar to the novel NW FET developed here as observed from Fig. 3a. Thereafter, Device G, H, I, J, K, L, M, N and O are designed keeping radial thickness of the strain silicon and strain SiGe layers at 1.5 nm while the source and drain radial thicknesses are varied between 3 to 10 nm but maintaining a 2 nm underlap length. As demonstrated in Table 2, increasing the source and drain diameters resulted in an enhanced device performance with increase in the device I_on_/I_off_ current ratio; however, extending the source and drain diameters beyond 16 nm has no positive impact due to saturation of I_on_/I_off_ current ratio on the device performance as revealed from Fig. 3b. Considering this aspect as attained from Fig. 3b of the device performance to the source and drain radial thickness, the current ratio relation is therefore calculated here for the first time for NW CGAA FET and is given as:

2 $$\frac{{I}_{on}}{{I}_{off}}={p}_{1}{\text{ln}}\left({r}_{SD}\right)+{p}_{2}$$

where r_SD_ is the radial thickness of the source/drain regions and p_1_ and p_2_ are the constants. As a result, the source/drain dimensions of 16 nm (Device L) is being considered as the optimized alternative and investigated for further device advancements. For better understanding, Fig. 2f shows a cross-sectional view of Device L. Device P is developed on introducing the stack gate high-k in the strained channel NW gate all around FET for the underlap length of 2 nm by growing 0.5 nm thick HfO_2_ high-k over a layer of 0.5 nm SiO_2_, so as to have the gate oxide thickness of 1 nm. The source and drain diameters are retained at 16 nm while thickness of st-Si and st-SiGe are kept at 1.5 nm each, the sectional view is shown in Fig. 2g. The stack compound of the high-k dielectric is determined for equivalent oxide thickness by:

**Table 1:** Devices developed and their dimensions.

Device	Device specification	Channel radial thickness (st-Si/st-SiGe) (nm)	Source/drain radial thickness (nm)
A	8 nm strained channel NW FET	1–1	2
B	**8 nm strained channel NW FET**	**1.5–1.5**	**3**
C	8 nm strained channel NW FET	1.5–2	3.5
D	8 nm strained channel NW FET	2–1	3
E	8 nm strained channel NW FET	2–1.5	3.5
F	8 nm strained channel NW FET	2–2	4
G	Device B with 2 nm gate underlap	1.5–1.5	3
H	Device B with 2 nm gate underlap	1.5–1.5	4
I	Device B with 2 nm gate underlap	1.5–1.5	5
J	Device B with 2 nm gate underlap	1.5–1.5	6
K	Device B with 2 nm gate underlap	1.5–1.5	7
**L**	**Device B with 2 nm gate underlap**	**1.5–1.5**	**8**
M	Device B with 2 nm gate underlap	1.5–1.5	9
N	Device B with 2 nm gate underlap	1.5–1.5	10
O	Device B with 2 nm gate underlap	1.5–1.5	11
**P**	**Device L with stack high-k gate**	**1.5–1.5**	**8**
**Q**	**Device L with stack high-k gate and high-k spacer**	**1.5–1.5**	**8**

Significant values are in bold.

**Table 2:** Comparision of on-current, off-current and current ratio of CGAA FETS.

Device	On-current (mA/μm)	Off-current (nA/μm)	I_on_/I_off_ (× 10^4^)
A	0.2	2.59	7.72
**B**	**1.35**	**2.09**	**64.59**
C	1.7	7.57	44.6
D	1.39	38	15.20
E	1.76	9.15	15.19
F	2.17	48	45
G	0.8	25	32
H	0.95	1.98	49
I	0.99	1.98	50
J	1.02	1.98	51
K	1.03	1.98	51.5
**L**	**1.04**	**1.98**	**52**
M	1.04	1.98	52
N	1.04	1.98	52
O	1.04	1.98	52
**P**	**1.19**	**0.33**	**352**
**Q**	**2.5**	**0.11**	**2134**
R^19^	1.2	1.2	100
S^23^	0.037	23	0.16
T^30^	1.57	5.5	28.54
IRDS 2025^25^	0.787	10	0.0787
U^34^	1	10	10

Significant values are in bold.

3 $$EOT=( \frac{3.9}{{K}_{high-k}}){t}_{high-k}$$

Furthermore, the gate stack device's overall equivalent oxide thickness is obtained as:

4 $${EOT}_{total}=(\frac{3.9}{{K}_{high-k}}){t}_{high-k}+ {t}_{ox}$$

where K_high-k_ is the permittivity of the high-k material, t_high-k_ is the thickness of high-k dielectric, and t_ox_ is the oxide thickness. The following provides the gate stack device's equivalent gate capacitance:

5 $$\frac{1}{{C}_{eq}}=\frac{1}{{C}_{high-k}}+\frac{1}{{C}_{ox}}$$

where the high-k capacitance is denoted by C_high-k_ and the silicon oxide capacitance by C_ox_.

The final novel Device Q is created as an advanced version of Device P where a 1 nm thick high-k (HfO_2_) corner spacer is employed to surround the 2 nm underlap length area while the stack high-k gate on the NW CGAA is created by placing 0.5 nm thick HfO_2_ (permittivity = 25) over 0.5 nm thick SiO_2_ as in Device P. Figure 2h depicts a cross-sectional view of Device Q. Figure 2i depicts the full 3D NW CGAA FET structure having enlarged Source/Drain regions, the underlap regions and the stack high-K while and Fig. 2j provides the channel cross section views of the novel Device Q. These newly developed and designed devices are analysed here while also compared with the existing CGAA Device R (Karbalaei et al. 22 nm stack high-k dielectric CGAA)^19^, Device S (Goel et al. spacer high-k gate underlap junction less CGAA FET)^23^, Device T (10 nm tri-layer strained channel CGAA FET)^30^, Device U (26 nm gate length vertically Stacked Gate-All-Around Si Nanowire CMOS Transistors)^34^, and the proposal of IRDS 2025^25^ of 2 nm technology node device. Devices R, S, and T are used in the current study for comparison validation and performance enhancement analysis. The experimentally developed Device U of Ritzenthaler et al.^34^ is used to calibrate the present NW GAA device for electrical properties. Table 2 details and presents all these devices (Device A to Device U) for the I_on_, I_off_ and the I_on_/I_off_ current ratio, which mostly determines the device performance. This evidently indicates that the novel NW FET developed as Device Q stands to be quite superior among all the 8 nm gate length devices designed here and fulfils the criteria of IRDS 2025 at the 2 nm technology node. Also to be noted that Device Q also surpasses the performances of the existing literature devices that being used in the resent days and the IRDS 2025 proposed device criteria. The strain channel is employed such that the quantum well barrier system is inlaid so as to entice energy band bending within the layers depicting carrier confinement in s-SiGe while enduring enriched electron flow via ballistic transport through the tensile strained silicon layer of the novel NW FET.

The Silvaco Atlas simulator results are calibrated by comparing them to the I_D_–V_GS_ plot in the literature of experimental data of Device U (Ritzenthaler et al.^34^). The I_D_–V_GS_ of the structure presented by Ritzenthaler^34^ (experimentally developed two-stacked Si GAA FET with a gate length of 26 nm) is generated at V_DS_ of 0.7 V and 0.05 V as reported, which almost accurately matches with the Silvaco simulation results on merging the Hansch quantum model with the Lombardi mobility model and is shown in Fig. 3c. Hence, the experimental Device U is utilized here to effectively calibrate the electrical properties of the novel GAA devices developed here. The device simulation parameters using Silvaco TCAD^35^ are calibrated to fit the experimental results, as acquired in Fig. 3c, which matches quite closely thus indicating that the simulation is performed quite precisely and efficiently.

Installing the strain technology in the channel region of CGAA FETs bandgap reduction due to strain engineering and carrier accumulation via ballistic transport happens within the channel, which is included in the overall examination of the strained channel CGAA. The novel device designed has the Type-II heterostructure system inducing the carrier confinement effect and the strain effect developing quantum well barrier in the region. This leads to the formation of a cylindrical Nanosystem with strained channel that sources ballistic transport phenomena across the extremely thin barrier layer. This sources the bandgap energy between the allowable energy levels in the reasonably thin region to widen, ultimately compensating the tensile strain and perhaps decreasing the SCEs with performance benefits.

The 8 nm CGAA (Device Q) source and drain areas are evenly doped with 5 × 10^19^ cm^−3^, whereas the channel doping is 10^15^ cm^−3^. For Devices Q, the diameters of source and drain are kept at 16 nm while the radial thicknesses of st-Si and st-SiGe layers are retained at 1.5 nm. The gate stack is produced by employing an interfacial oxide layer in combination of SiO_2_ and HfO_2_ (permittivity k = 25) of 0.5 nm each surrounded over the channel, providing the device with a higher physical gate height. The novel NW FET device developed appears to be enhanced than the IRDS 2025^25^ prediction of the 2 nm technology node device due to the incorporation of the strain channel engineering, stack high-k gate and high-k spacer with gate underlap. Meshing, material filling, and doping are the three major phases in the fabrication process of an 8 nm strained n-channel CGAA having stack high-k gate. Based on the material being employed in the specific portions of the created meshes, the device structure is separated into distinct zones. In the device, dopant is silicon, silicon oxide an interface layer, high-K (HFO_2_) gate dielectric and also as a spacer, and polysilicon as a gate contact. Figure 3d depicts the probable fabrication process flow for the device. Epitaxial growth is used to build the channel area, resulting in a two-layer stressed Nanosystem. The channel comprises of a multilayer system with the outside layer being silicon and the inner layer is Si_1−x_Ge_x_ with a mole percentage of x = 0.4 grown to fill the inner cylindrical space. The strained hetero-channel region is formed by covering the inner SiGe region with an upper layer of Si thus forming quantum well barrier nanosystem channel.

The total strain in the ultra-thin strained channel NW CGAA FET is calculated to be:

6 $${\Psi}_{ch{-}strain}=\sum \pi.{\fancyscript{f}}({R}_{eff}{)}^{2}$$

7 $${R}_{eff}=\frac{{r}_{ou s{-}Si }}{{r}_{ou s{-}Si} - {r}_{in s{-}SiGe}}$$

where r_ou s–Si_ is the radius of the outer st-Si and r_in s-SiGe_ is the radius of the st-SiGe, while R_eff_ is the effective radius of the novel CGAA device developed here based on the radial thickness of the cylindrical channel embracing the two ultra-thin layers (outer and inner st-Si and st-SiGe layers respectively) that forms a 2D circular bi-layered nano channel as illustrated in Fig. 1b. Ψ_Ch-strain_ is the overall strain developed here as the function of R_eff_, which is formed in 2D throughout the length of the channel of the device creating the cylindrical GAA FET. Because of device symmetry, the Poisson's equation of the strained channel CGAA FET is stated as:

8 $$\frac{1}{x}\Delta \left(x.\Delta \Phi \left(x,z\right)\right)+{\Delta }^{2} \Phi \left(x,z\right)={qN}_{A}/{\mathtt{P}}_{s-Si}0\le \mathrm{ x }\le \mathrm{ R}, 0\le \mathrm{ z }\le {\text{Lg}},$$

where Φ(x,z) is the electrostatic potential in the channel, P_s-Si_ is st-silicon permittivity, R is the radious of the device, L_g_ is the gate length of the device. A parabolic approximation of the ultra-thin channel potential distribution in the radial direction is used to solve Eq. (8), and it is given by:

9 $$\Phi \left(x,z\right)={A}_{0}\left(z\right)+{A}_{1}\left(z\right)x+{A}_{2}\left(z\right){x}^{2}$$

where the coeffiecients A_0_(z),A_1_(z), and A_2_(z) are functions of channel length in 3D, which is quantified considering the boundary conditions for the novel device:

i. With the assumption that the gate oxide and outer st-silicon interfaces have an electrostatic surface potential interface to be:

10 $$\Phi \left(x,z\right){|}_{x=R}={\Phi }_{s}\left(z\right)$$

ii. Inner s-SiGe forms a well at the device centroid, with hole being the predominant carrier, resulting in negligible carrier density with zero electric field, as given by:

11 $$\Phi \left(x,z\right){|}_{x=0}=0$$

iii. The electric field at the intersection between the gate oxide and the outside st-Silicon is continuous and is given as:

12 $$\Delta \left(x,z\right){|}_{x=R}=\frac{{C}_{eq}{\prime}}{{\mathtt{P}}_{st-Si}}\left[{V}_{GS}-{\left({V}_{FB}\right)}_{st{-}Si}-{\Phi }_{s}\left(z\right)\right]$$

The coefficients are found and so adjudicated as follows after applying the boundary conditions Eqs. (10) and (11) in Eq. (8):

13 $$\Phi \left({\text{x}},{\text{z}}\right)={\Phi }_{{\text{s}}}\left({\text{z}}\right)-\frac{{{\text{xC}}}_{{\text{eq}}}{{^{\prime}}}}{2{{\mathtt{P}}}_{{\text{st}}{-}{\text{Si}}}}\left[{{\text{V}}}_{{\text{GS}}}-{\left({{\text{V}}}_{{\text{FB}}}\right)}_{{\text{s}}{-}{\text{Si}}}-{\Phi }_{{\text{s}}}\left({\text{z}}\right)\right]+\dots \frac{{{\text{C}}}_{{\text{eq}}}^{{^{\prime}}}}{2{R{\mathtt{P}}}_{{\text{s}}-{\text{Si}}}}\left[{{\text{V}}}_{{\text{GS}}}-{\left({{\text{V}}}_{{\text{FB}}}\right)}_{{\text{st}}{-}{\text{Si}}}-{\Phi }_{{\text{s}}}\left({\text{z}}\right)\right]{{\text{x}}}^{2 }$$

The electron is the dominant carrier transport factor in this st-channel, however the hole is the majority carrier in the inner-SiGe layer, so the electron flow is minimal, and the potential is ignored in inner st-SiGe. The distribution of the electric field is estimated along the z axis as:

14 $${\rm E}[\left(x,z\right)=-\frac{d\Phi \left(x,z\right)}{dx}=\frac{{C}_{eq}{\prime} .x}{R. {\mathtt{P}}_{st{-}Si}}{\Phi }_{s}\left(z\right)$$

where gate oxide capacitance, c_eq_is further given as ${c}_{eq}{\prime}=\frac{{\mathtt{P}}_{eq}}{R{\text{ln}}\left(1+\frac{EOT}{R}\right)}$ . Surface potential at gate-oxide contact is Φ_s_ (z), and ${\mathtt{P}}_{\text{eq}}$ represents the equivalent gate oxide permitivity, which is the sum of permitivity of SiO_2_ and HfO_2_., EOT is equivalent oxide thickness. On acquiring the strain effect for the novel NW FET, the threshold voltage of the 8 nm is given by:

15 $${{V}_{th}}_{st{-}si}=\frac{\left|{Q}_{SD}{{^{\prime}}}(max)\right|}{{C}_{eq }}+{{V}_{FB}}_{st{-}si}+2{{\psi }_{fn}}_{s-si}$$

where $${\left({V}_{FB}\right)}_{st-Si}={\left({V}_{FB}\right)}_{si}+\Delta { V}_{FB}$$ , (V_FB_) = φ_M_ − φ_si_ and $$\Delta {V}_{FB}=-\frac{(\Delta {E}_{c}{)}_{s{-}Si} }{q}+\frac{(\Delta {E}_{g}{)}_{s-Si}}{q}-{V}_{T }{\text{ln}}\left(\frac{{N}_{Vsi}}{{N}_{Vst{-}Si}}\right)$$.

The strained silicon and silicon flat-band voltages are (V_FB_)_st-si_ and (V_FB_)_si_, respectively. The symbol φ_si_ represents the silicon work function, whereas φ_M_ stands for metal work function. The effective density of states of silicon and strain silicon for the valence band are N_vsi_ and N_vs-si_, respectively. V_T_ = kT/q is the thermal voltage, ${Q}_{sd}{\prime}\left({\text{max}}\right)=e{N}_{d}{x}_{dt}$ , ${{\psi }_{fn}}_{st{-}si}={V}_{T}{\text{ln}}\left( \frac{{N}_{D ch}}{{n}_{ st{-}si}}\right)$ , the space charge width is given by ${x}_{dt}$, the doping concentration of donor in strained channel Nanosystem is given by N_Dch_, and carrier concentration of outer s–Si layer is denoted by n_st-si_. The threshold voltage is calculated from Eq. (8) for the newly developed Device Q and is acquired to be 0.34 V voltage. The Silvaco tool is utilised to do the analysis of the novel 8 nm strained channel NW FETs. Several models are employed to run the simulation and attain the parameters required to attain the device performance. Models of recombination and Augur model incorporate the current density effect in the device, while the Shockley–Read–Hall (SRH) model incorporates generation and recombination of carriers. As the device developed here is in the nanoscale regime with several ultra-thin layers in the channel region forming quantum well barriers, quantum effects in both the transverse and transport directions plays a significant role in determining device properties while due to wave particle duality effect of electrons in the channel layers the carrier transport phenomenon acquires an adverse effect. To meet the requirement the Non Equilibrium Green's Function (NEGF) model is employed for indigenous calculations of the carrier transport. The overall current produced by the NEGF solution agreed with the sum of the currents through the device at each point taken. The quantum model in addition to providing a quantifiable assessment of the likely enhancements in mobility, electron velocity and electrostatic potential within the device is the Hansch's quantum model that takes into account the carrier confining phenomena in the channel inversion layers. The device's mobility analysis is incorporated through the Lombardi CVT model. The Band-Gap-Narrowing (BGN) Model takes into consideration the effects of band narrowing in a channel caused by induced strain. The newly developed 8 nm CGAA devices are next subjected to qualitative and quantitative investigations, and estimates are made for the SCEs such as SS and DIBL and the output performance based on I_on_/I_off_ ratio and drive current across the channel are analysed while the quantum effects are discussed further.

## Performance analysis and discussion

**Figure 4:**
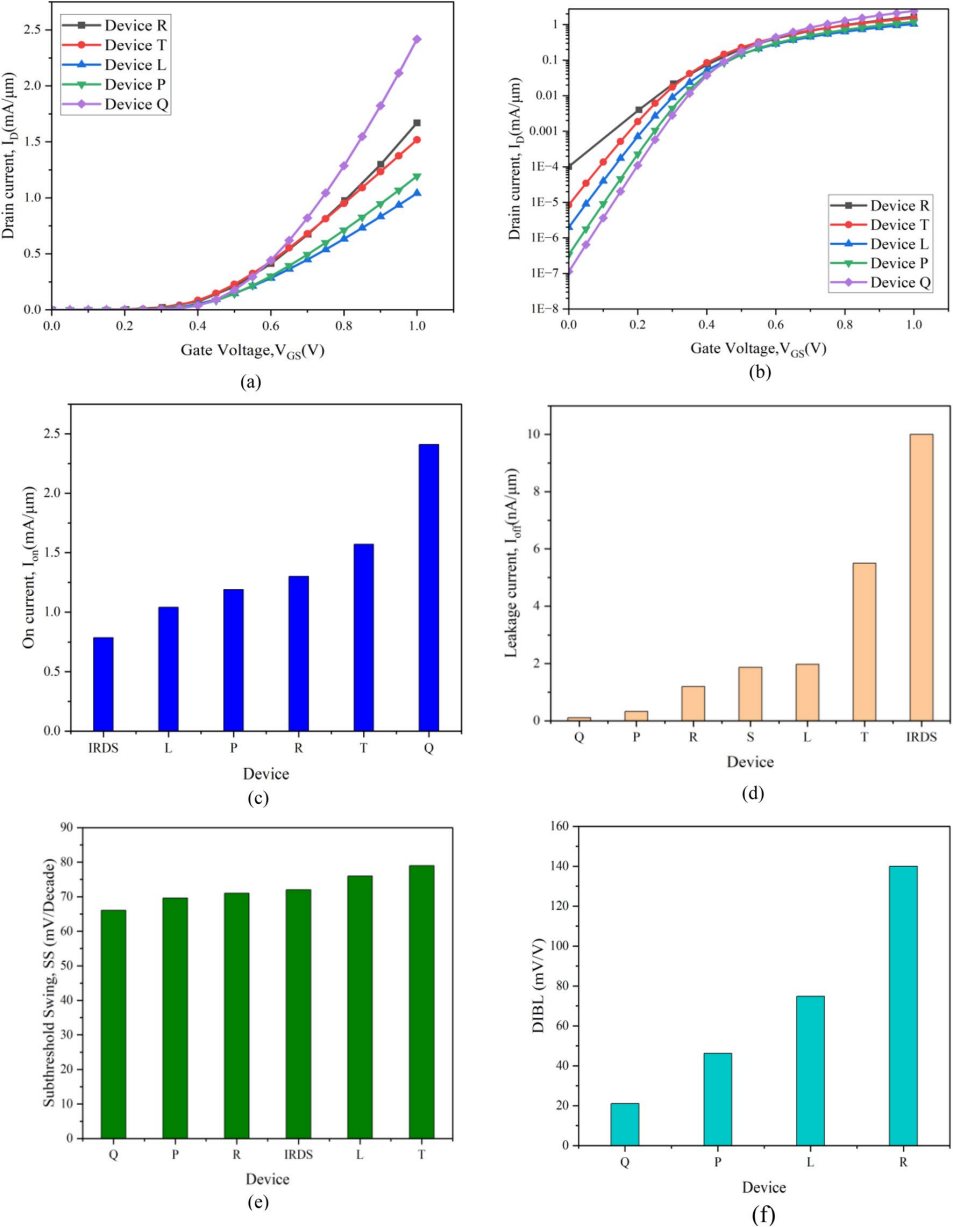
(**a**) Transfer characteristics (linear) of I_D_–V_GS_ at V_DS_ = 1 V for device L, P, and Q are compared with existing device T and device R. (**b**) The transfer characteristics (logarithmic) fussing the leakage current. Improved performance in both cases for device Q is apparent. (**c**) On-current of the present device L, P, and Q are compared with the proposed data of IRDS 2025 at 2 nm technology node and existing device R and device T, (**d**) comparison of leakage current of device L, P, and Q with the existing device R, device S, device T and IRDS 2025 at 2 nm technology node, (**e**) Subthreshold swing for device L, P, and Q are compared with existing device R and device T and IRDS 2025 at 2 nm technology node, (**f**) DIBL comparison of the device L, P, and Q with existing device R.

The effects of gate underlap and induction of the high-K material on various physical parameters of the newly developed 8 nm strained channel NW FETs are analyzed. Various significant parameters are studied by taking into account the stack high-K gate and gate underlap while also the increase in the source and drain radius of the developed devices such as drain current vs gate voltage (I_D_ − V_GS_), On-current, Off/leakage-current, the On–Off current ratio and the transconductance. The ultra-thin strained channel (st-Si/st-SiGe) system with 1.5 nm thicknesses are used in the development of the novel 8 nm gate-all around NW FET with stack high-k gate (Device Q), which is described in Table 1. Device Q comprises of a gate stack of 0.5 nm thin high-k grown on top of the 0.5 nm SiO_2_ interfacial layer and a high-k spacer surrounding the underlap region of 1 nm thick. This construction ensures that the gate stack in Device Q minimizes the impact of threshold voltage-offset, lowers short channel effects, and enhances the I_on_/I_off_ ratio. Device L is a strained channel NW FET with a 2 nm underlap for the 8 nm channel length device and Device P is the 8 nm channel length NW FET with stack high-k gate. Device R (22 nm with stack high-k gate CGAA FET)^19^, Device S (high-k spacer with underlap junction less GAA FET)^23^, and Device T (Type-II hetero strained channel CGAA FET with a 10 nm gate length)^30^ are references for further research and analysis of the current work. For the devices developed here, I_D_-V_GS_ characteristics are presented in Fig. 4a. The data makes it quite evident that, in comparison to all other devices, on-current of Device Q is higher at a given gate voltage. Thus it can be concluded from the fact that the high-k dielectric material has enhanced the capacitance, which raises charge as (Q = CV) and in-turn the current also increases. Further band-lowering in Device Q due to its underlap region is caused by the additional high-k spacers on source and drain side. Since high-k spacers improve the terminating fringing field convergence into the gate-source and gate-drain regions, it is possible to deduce that the devices eventual barrier potential will be smaller and thereby the Fig. 4b with the log scale plot invariably signifies lowest off-current in contrast to all others including the existing devices. The quasi-ballistic carrier transit event and the nutrition obtained from the trapped carriers of the strain affected quantum wells of the ultrathin st-Si layers in the Nanosystem device serves to validate this demonstration. Thus, a unique phenomenon of the quantum carriers succumbing with charge centroid being formed leads to a superior representation in the novel Device Q. This ascribes to the installing of the Type-II heterojunction system in the narrow channel of the nano-device. Also, to be noted that this is the first of its kind to implement the high-k spacer and the stack gate high-k with the hetero-strain channels as two methods for controlling SCEs at nano regime.

Figure 4c shows the ON current (I_on_) analysis for Devices L, P, Q, R, and T, which are conformed as 1.04 mA/μm, 1.19 mA/μm, 2.4 mA/μm, 1.2 mA/μm, and 1.57 mA/μm, respectively. The graph demonstrates that Device Q outstrips the four other devices, proving itself to be the most dominant gadget at the time. To that end, a high-k spacer of 8 nm gate stack CGAA NW FET at 8 nm length (Device Q) confirmed an emphatic enrichment of 101% above Device R^19^, 53% above Device T^30^, 150% above silicon channel CGAA FET^14^, and 206% above the IRDS 2025 proposal of the 2 nm technology node are unquestionably an incredible attainment as also revealed in Table 3. In Device Q, the high-k dielectric material increases the capacitance, which raises charge and thus increases current; additionally, the ballistic transport through the channel due to hetero-strain effect and higher fringing field owes to the spacer high-k. This increases carrier concentration on the channel subsequently negligible scattering mechanism is experienced resulting in greater carrier mobility and thereby improved I_on_ is perceived. The Off-current (I_off_) evaluation is performed for the developed devices L, P, and Q, which are calculated to be 1.98 nA/μm, 0.33 nA/μm, and 0.11 nA/μm, respectively, and compared with IRDS 2025, Device S, and Device T, and are presented in Fig. 4d. The data undoubtedly illustrates Device Q to have the minimal leakage current when compared to the other five devices, showing that it is the best device at the given regime. The leakage current in Device Q is reduced mainly due to the introduction of the gate underlap being sandwiched by the high-k spacer in the device. The use of high-k spacer materials reduces the off-current due to increased electrostatic barrier potential encountered by the carriers and increased channel parasitic resistance due to gate underlap.

**Table 3:** Comparision OF electrical parameters of proposed device with other CGAA FET.

Device	On-current (mA/μm)	Off-current (nA/μm)	I_on_/I_off_ (× 10^4^)	Maximum transconductance g_mmax_ (ms/μm)	Subthreshold swing (mV/decade)	DIBL (mV/V)
**Q**	**2.5**	**0.11**	**2134**	**4.11**	**66**	**21**
R^19^	1.2	1.2	100	2.5	71	Not given
S^23^	0.037	23	0.16	0.006	0.07	Not given
T^30^	1.57	5.5	28.54	2.11	79	Not given
IRDS 2025^25^	0.787	10	0.0787	1.6	72	Not given
U^34^	1	10	10	Not given	68	60
22 gate length Silicon channel CGAA FET^14^	1	10	10	1.8	50	Not given
GAA TFET^21^	0.0000008	0.00001	8	Not given	144	Not given
28 gate length NW GAA MOSFET^20^	0.003	0.8	0.375	18.1	Not given	Not given
MBFET^26^	Not given	Not given	Not given	Not given	65	20

Significant values are in bold.

Short Channel effects (SCEs) such Subthreshold Swings (SS) and DIBL are also analyzed. SS for the developed devices L, P, and Q are detected to be 76 mV/decade, 69 mV/decade, and 66 mV/decade, respectively, and are compared with the 2 nm technology node IRDS 2025^25^, Device R^19^ and T^30^ as depicted in Fig. 4e. The Device Q is outwardly observed from Fig. 4e to have a lower Subthreshold Swing among all the five other devices compared here. This is attributed to the fact that the high-k spacer causing band-lowering in underlap region of Device Q, thereby Device Q can switch faster and also because high-k (HfO_2_) stack gate the leakage through the gate is reduced. DIBL for the developed devices L, P, Q are calculated to be 74.78 mV/V, 46.24 mV/V, and 21.06 mV/V, respectively and are compared with existing 22 nm stack high-k gate CGAA FET (Device R) and is depicted in Fig. 4f. The DIBL and SS of the Device Q are approximately equal to those of Samsung's Multi-Bridge Channel FETs^26^. The DIBL is calculated as:

16 $$DIBL=\frac{({V}_{th1}-{V}_{th2})}{({V}_{DS2}-{V}_{DS1})}$$

where V_th1_ and V_th2_ are threshold voltages calculated at different drain bias of V_DS1_ = 0.1 V and V_DS2_ = 1 V respectively.

**Figure 5:**
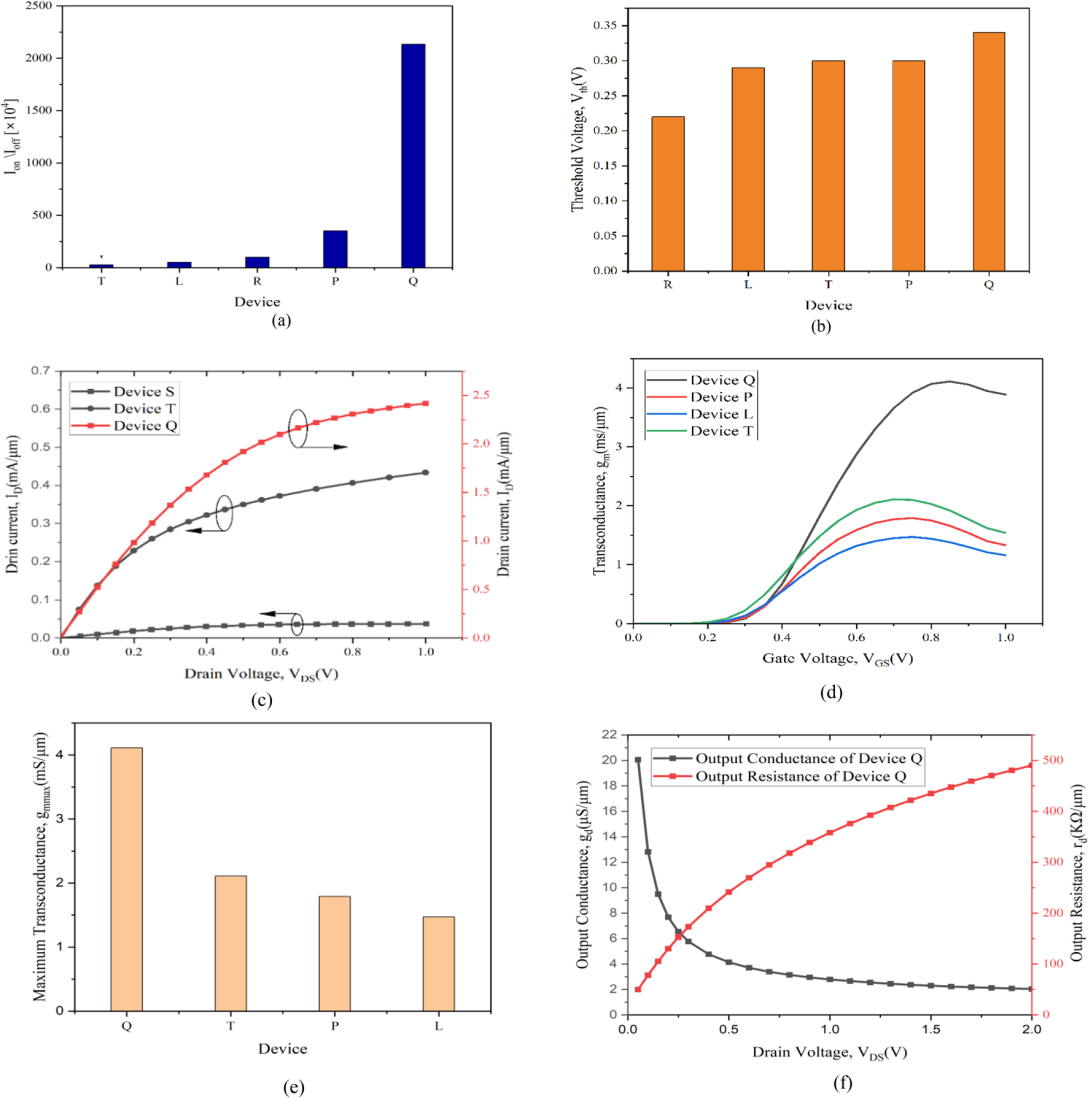
(**a**) Comparison of I_on_/I_off_ ratio developed devices with existing device R, device T and IRDS 2025, (**b**) threshold voltage comparison of developed devices with existing device T and device R, (**c**) I_D_–V_DS_ characteristic at V_GS_ = 1 V validating device Q with existing device S and device T, (**d**) transconductance (g_m_) comparison of developed devices with existing Device T. (e) Maximum transconductance (g_m max_) comparison providing healthier performance for device Q over device T, (**f**) output conductance (g_d_) and output resistance (r_d_) of the device Q.

Figure 5a depicts I_on_/I_off_ ratio for Devices L, P, Q, R and T. The Device Q, as evident from Fig. 5a, poses to be a superior Device in comparison to Device L, P, R and T due to its better control over the channel. The spacer high-k with the gate underlap assembly provides healthier gate control enhancing the on-current while it also reduces the leakage current, thus contributing to the enrichment I_on_/I_off_ current ratio. Hence, Device Q warrants being an improved device for faster switching and digital-applications. The threshold voltages of Devices L, P, Q, and R^19^ are determined to be 0.29 V, 0.30 V, 0.34 V and 0.22 V, respectively, from the derived Eq. (8), where Device Q is observed to be higher than the existing Device T^30^ due the incorporation of the high-k spacer. The acquired threshold voltages are thereafter plotted for comparison as shown in Fig. 5b and is evidently observed that V_th_ for the Device R is lower, but off-current and SS of Device R is higher indicating Device R is not a well performing device. While Device Q gives better I_on_/I_off_ ratio along with reduced leakage and SS so the device stands as the substitute device and is the expected future device of choice.

Figure 5c depicts the I_D_-V_DS_ characteristics as obtained and the novel NW FET developed here Device Q is compared with the existing devices of the literature Deice S and Device T. A detailed comparison of the novel Device Q is presented in Table 3 with the existing literature CGAA FETs and Device Q having the strain ultrathin channel Nanosystem technology with stack high-k gate and underlap spacer wrapped high-k is observed to outshine all others. Based on the analysis observed due to the inculcation of the strain engineering in the NW FET (Device Q) forming quantum well barrier system with the heterostructure having Type-II band alignment and the high-k spacer sandwiching the underlap to reduce the parasitic effects much superior characteristics is attained with massive enhancements in contrast to the existing device designs. The combined use of a high-k spacer with a strain channel enhances carrier concentration on the channel, results in such an emphatic increase in carrier mobility and enhanced drain current as in Fig. 5c.

The transconductance (g_m_) analysis of the 8 nm channel length novel NW device is depicted in Fig. 5d, which provides the vital assessment for authenticating the performance and switching speed of the n-channel device and is calculated by:

17 $${g}_{m}=\frac{\partial {I}_{D}}{\partial {V}_{g}}$$

The plot for the NW strained channel CGAA FET showcases that g_m_ of Device Q is higher than Device L and P. This is due to the fact that the high-k spacer in the device is having higher permittivity and is able to subdue the parasitic resistance in the device deliberating higher transconductance. Hence, the maximum transconductance (g_m max_) of Device Q is calculated to be 4.11 mS/m at V_GS_ = 0.7 V, which is also compared with the similar existing devices of L, P and T and is observed to be higher than the devices as revealed from Fig. 5e. The higher the device transconductance, the greater is the gain of the device; hence Device Q is much healthier when applied for faster switching. Furthermore, the strained channel system induced in the novel NW FET results in the creation of the quantum wells with Type-II heterostructure band alignment at nano regime, which stirs an improvement in carrier mobility due to the amalgamation of ballistic transport and quantum carrier confinement in the channel area. As a result, the surface-parallel carrier transport characteristics of the device progresses further resulting in improved transconductance. Thus, the gate stack strain channel CGAA with high-K spacers (Device Q) is a superior device, and thereby predicts a possible rise in the drain current for strained channel CGAA NW FET. This in turn instigates faster switching speed for the novel device hence additional RF/AF parameter analysis is conducted so as to have Device Q applicably ready for future design based CMOS circuitry. The output conductance, g_d_, and the output resistance, r_d_, for the NW strained channel CGAA with high-k gate stack and spacer are calculated by:

18 $${g}_{v}=\frac{{I}_{d}}{{v}_{d}}$$

19 $$\mathrm{and }\, {r}_{d}=\frac{1}{{g}_{d}}$$

These quality measurements directly affect the intrinsic gain of the NW channel in the device and therefore provide an understanding of Device Q which will have effects when employed in CMOS circuitry systems. The determined output conductance of the device is in μS/μm for the channel region and results in very high output resistance in the range of KΩ, as attained in Fig. 5f, thus providing a significantly amplified intrinsic gain. The output conductance quickly reaches linearity, ensuring increased driving proficiency at lower drain voltage, a well-known occurrence for superior FETs and is clearly realized in the novel Device Q reassuring the device to be suitable for faster switching operation ready for implementation in RF/AF based CMOS circuitry and hence meets the desired requirement for the purpose the novel device is designed and explored.

**Figure 6:**
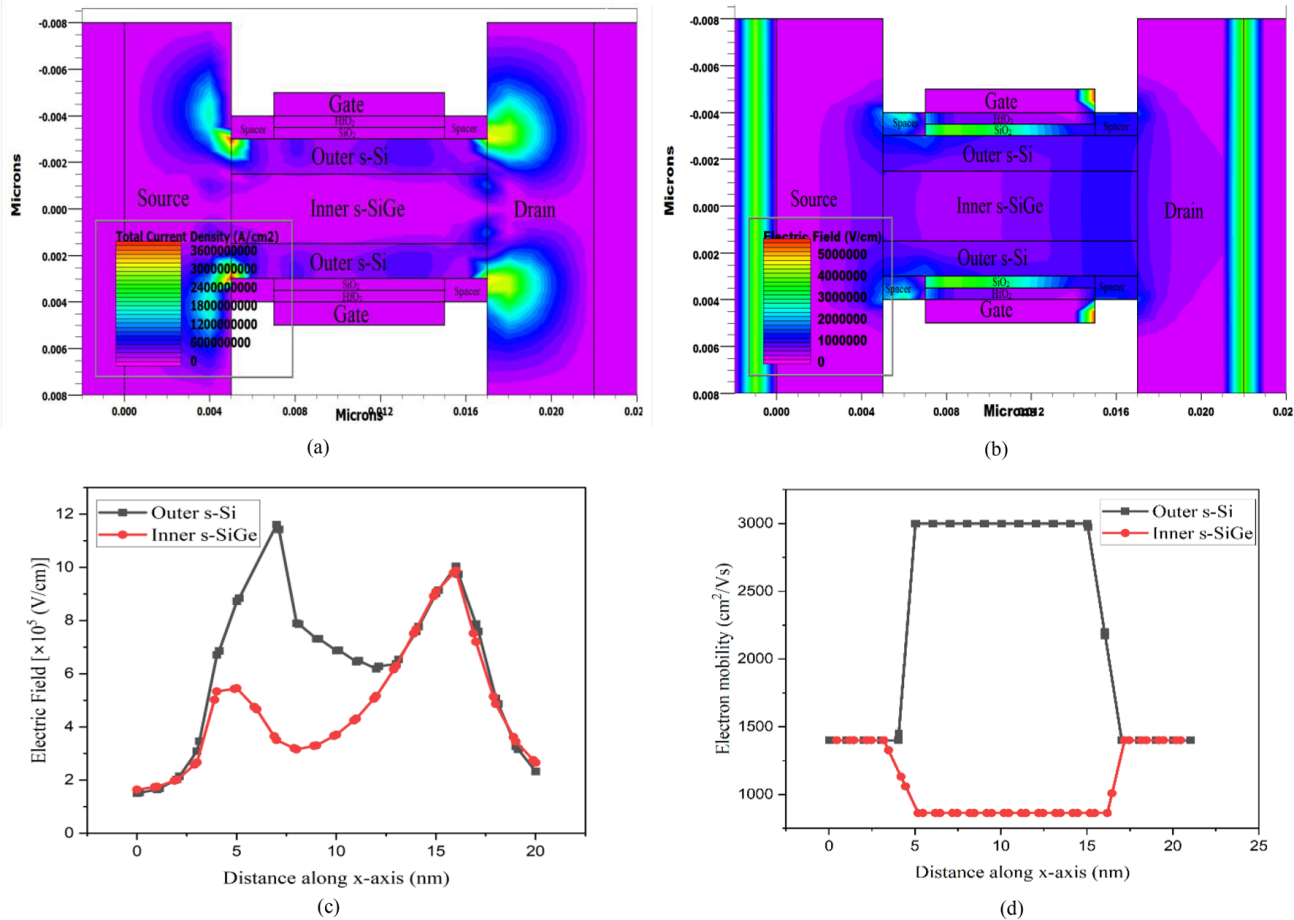
(**a**) Source-channel and drain-channel interfaces vehemently shows the current density contours as observed for device Q, (**b**) electric field contours displaying source-channel and drain-channel interfaces with higher E-field for device Q. (**c**) Electric filed graphical presentation of device Q indicate peaks at soruce-channel and drain-channel interfaces. (**d**) Electron mobility with negligible varation in channel observed for device Q.

Considering the understanding it is evidently observed that Device Q stands as the device to meet and surpass the proposed requirement of IRDS 2025 with 2 nm technology node and is full on to meet the requirements of the future 1 nm node of IRDS 2028^25^. Further to this affect, an in depth analysis of Device Q is explored here. Figure 6a analyses the fluctuation in current density across the Device Q. The source-channel interface and the drain-channel interface of the outer st-Si layer experiences significant variation in current density. The interface corners are observed to be prone to quite high current density due to less carrier movement while the outer st-Si is detected to provide an average current density throughout. The dual influence of the Type-II band alignment as well as the stack high-k dielectric for the st-Si channel device results in electronic carrier confinement in the channel. Because SiGe is highly susceptible to hole carrier movement, the outer st-Si layer experiences increased electron flow via ballistic transport. Also it is to be noted that as the capacitance of the fringing fields are increased by high-k spacers, there is a large increase in current density at the source-channel and drain-channel interfaces.

Figure 6b demonstrates the electric field (EF) variations across Device Q. Because the high-k spacer material has increased fringing field capacitance, which increases the charge, apparently greater electric field is created as revealed near the source-channel and drain-channel interface as is also clearly detected from the graphical interpretation of Fig. 6 (c). Furthermore, the evaluation shows that the EF is stronger at the underlap region than at the outer st-Si layer channel due to the influences of the high-k spacer, gate stack and Type-II hetero band-alignment, thereby promoting carrier movement through the st-Si layer of the channel.

The change in electron mobility in Device Q is observed from Fig. 6d. The strain effect of the channel, ballistic carrier transport, and generation of higher fringing field due to the introduction of the high-k spacer, increased carrier concentration on the channel is inevitably detected leading to increase the carriers via succumbing of quantum charge carriers in the channel area. This results in negligible scattering effects within the ultra-thin channel and increased carrier mobility in the st-Si channel layer. The mobility is observed to stay steady across the channel region showing minor scattering influences that leads to augment the device performance. Since SiGe is highly susceptible to hole carrier movement the electron mobility is low in the inner st-SiGe layer.

**Figure 7:**
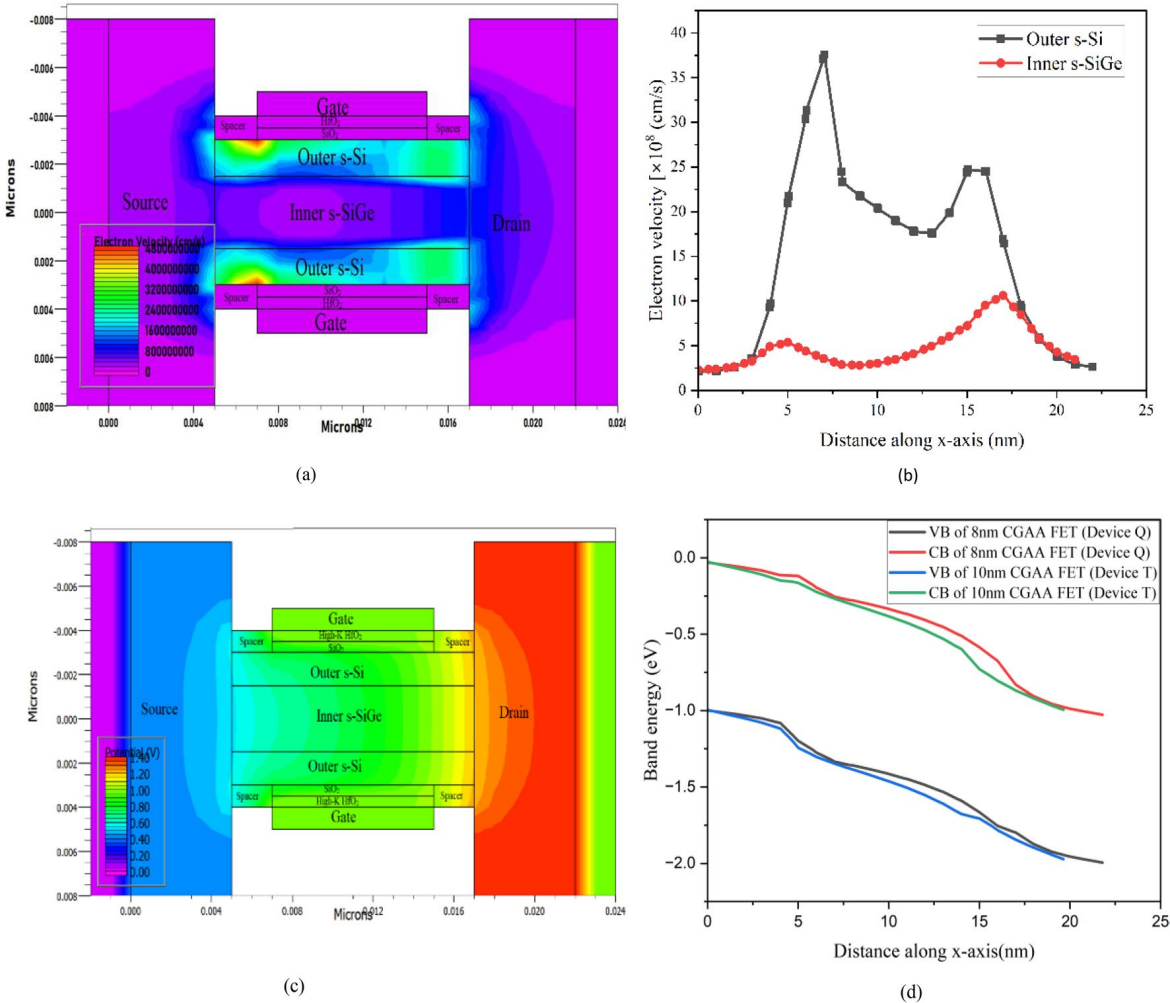
(**a**) Electron velocity contours observed across device Q, (**b**) electron velocity graph of device Q revealed higher electron velocity in s-Si layers along the NW channel, (**c**) electrostatic potential contours observed across the device Q, (**d**) band energy diagram comparing 8 and 10 nm CGAA FETs along the length of the channel (through the st-silicon layer) at the On state.

The electron velocity over Device Q is depicted in Fig. 7a, b. The outer s–Si layer of the device channel is observed to have significantly higher electron velocity than the inner s-SiGe barrier layer and this is directly attributed to the fact that the channel is having tensile strain effect on the Si layer forming the ultra-thin layer to be the quantum well in region. Since SiGe barrier layer is more susceptible to hole flow, the electron velocity in s–Si layers is exceptionally higher than in s-SiGe layer. Furthermore, the band is lowered in the gate to source/drain underlap regions due to the sandwiching by the high-k spacer, causing the carrier velocity to increase in the region to sabotage the parasitic resistance to some effect. On examining the electrostatic potential contours as observed in Fig. 7c of the novel CGAA NW FET channel, it follows quite similarly to the electric field and electron velocity analysis, which gradually increases through the channel region from source-end to drain-end and depends on the conveyance of carriers through the region. This is also well supported and observed from the electric field and the electron mobility analysis performed in Fig. 6c, d, respectively. This is because of the high-k spacer in conjunction with the strained channel that an enhanced conduction path in created once the gate bias crosses the threshold voltage and the ballistic carrier transport is incurred along the length of the channel due to the generation of higher fringing fields in the region. Figure 7d depicts the energy band diagram for state variations of 8 nm and 10 nm CGAA FETs (Device Q and T) along the x-axis across the s–Si layer of the channel. The channel radial thickness of the 8 nm device being less in comparison, higher quantum carrier confinement in the Type-II energy levels are expected in Device Q, which will shift the energy states to higher levels hence, higher threshold voltage is expected as acquired in Fig. 6b. Based on this aspect the device performance for Device Q is expected to degrade slightly, but as both these devices at nano-dimensions endure ballistic transport via the s–Si layers only so the quantum carriers transport through the continuum of band without undergoing scattering effects. Also as observed from Fig. 7d, the NW devices undergoes a reduction in band energy occurs towards the drain end due to the application of a high drain voltage, which significantly inhibits electron tunnelling between the valence and the conduction bands enriching the current. For Device Q however, the combined effect of the high-k spacer and the underlap region with strain increases the electron mobility through the channel thereby leading to the formation of the quantum well barrier system in the heterostructure channel of the NW FET that instigates the enhancement in carrier velocity of the device and thus the drive current is augmented while the SCEs are sabotaged inducing enough elemental band splitting in the region inculcating ballistic transport through the channel of the device.

**Figure 8:**
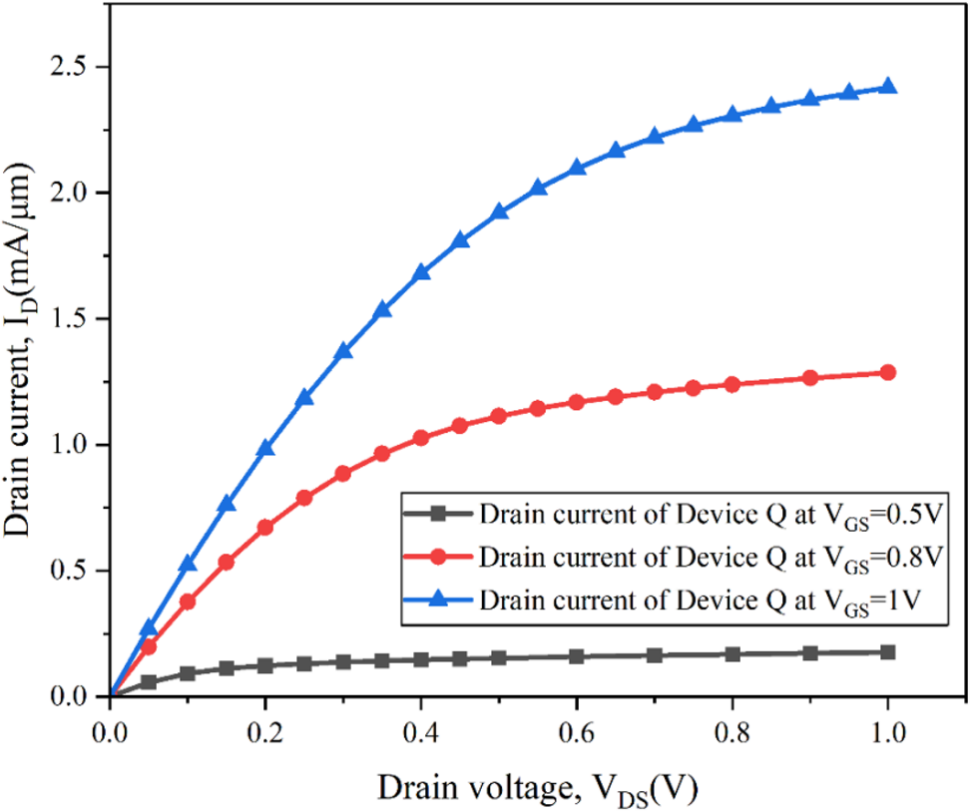
I_D_–V_DS_ characteristic at various V_GS_ = 0.5 V, 0.8 V and 1 V validating the output performance of device Q.

The investigation of Device Q is henceforth laid here based on the output performance, which is distinguishable from the I_D_-V_DS_ characteristic that is depicted in Fig. 8. The figure clearly indicates that due to the attributions of the novel architecture that Device Q inherits with the combined effect of high-k spacer, stack high-k gate and the strain induced quantum well barrier system in the channel through Type-II band alignment it has much superior characteristics which is also compared to the existing designs as observed in Fig. 5d. Considering this aspect Device Q is further analysed here for the output performance at different V_GS_ of 0.5 V, 0.8 V and 1 V in Fig. 8. The combined use various atrocities Device Q is observed to have increased carrier mobility and enhanced drain current.

The results show that in Device Q strained channel CGAA NW FET architecture is conceptualized with strain engineering which also employees high-K spacer and stack high-k gate resulting in drive current to increase while the leakage current and related SCEs are degraded to a huge extent crediting to negligible current crowding and scattering effects. The I_on_/I_off_ ratio, transconductance and conductance improves a large degree with electron mobility and current density in the channel to be overwhelming because of the heterostructure based quantum well-barrier in the channel of the device and thus augmenting the overall device performance.

## Conclusion

The 8 nm gate underlap strain channel Nanowire (NW) FET with high-k spacer sandwiching the underlap and stack high-k gate architecture (Device Q) is developed here for the first time and is explicitly analyzed for device performance. Considering the strain effect and high-k insertions the threshold voltage of the device is calculated based on the derivation done. The novel Device Q is then analysed and compared for the electrical performance characteristics with the existing strain channel CGAA (Device T), High-k gate stack CGAA FET (Device R), gate stack underlap CGAA MOSFET (Device S), and the proposed device consortium from IRDS 2025 for the 2 nm technology node. In terms of switching speed, leakage (off) current, on-current, subthreshold swing, and DIBL, Device Q is observed to surpasses the existing High-k CGAA FETs and High-k spacer-based gate stack MOSFETs. The ultra-thin strained channel (st-Si/st-SiGe) with High-k spacer and gate stack (Device Q) based device includes Type-II hetero-band alignment forming a quantum well barrier system, which leads to carrier mobility enhancement through ballistic transport and quantum carrier confinement in the channel region of the device. Device Q provides highly improved I_on_/I_off_ ratio performance with ON current enhancement of 100.8% when compared to the 22 nm High-K CGAA FET (Device R), and an ON current enrichment of 53% when compared to the 10 nm strain channel CGAA FET (Device T). The device is also observed to provide boosted carrier mobility and splendid electron velocity. As a result, Device Q developed here delivers superior on-current, transconductance, switching speed, and leakage current, and reduced SCEs leading to augmented overall device performance. The device also is far superior in comparison to the proposals of IRDS 2025 of the 2 nm technology node data. Hence, the device is particularly suitable for faster switching speed based digital applications and is the future device of choice.

## Data Availability

All data generated or analysed during this study are included in this published article, and can be requested from the corresponding author on reasonable request.

## References

[1] Pham-Nguyen, L., Fenouillet-Beranger, C., Ghibaudo, G., Skotnicki, T. & Cristoloveanu, S. Mobility enhancement by CESL strain in short-channel ultrathin SOI MOSFETs. *Solid-State Electron.* **54**, 123–130 (2009). 10.1016/j.sse.2009.12.006

[2] Riyadi, M. A., Saad, I. & Ismail, R. Investigation of short channel effect onvertical structures in nanoscale MOSFETs. *Telecommun. Comput. Electron. Control* **7**, 175–180 (2009).

[3] Seoane, N., García-Loureiro, A. & Kalna, K. Special issue: Nanowire field-effect transistor (FET). *MDPI J. Mater.* **13**, 1845 (2020). 10.3390/ma13081845 PMC7215818 32295217

[4] Mohsenifar, S. & Shahrokhabadi, M. H. Gate stack high-κ materials for Si-based MOSFETs past, present, and futures. *Microelectron. Solid State Electron.* **4**, 12–24 (2015).

[5] Panti, D., Priji, Z. & Pavlovi, Z. Process design and optimization of the channel doping profile in power VDMOSFETs. *Microelectron. J.* **27**, 191–200 (1996). 10.1016/0026-2692(95)00088-7

[6] Ying, S., Xiao, Y., Rui, Z., Bing, C. & Ran, C. The past and future of multi-gate field-effect transistors: Process challenges and reliability issues. *J. Semiconduct.* **42**, 1674–4926 (2021)

[7] Choi, S., Sun, W. & Shin, H. Analysis of stress-induced mobility enhancement on (100)-oriented single- and double-gate n-MOSFETs using silicon-thickness-dependent deformation potential. *Semicond. Sci. Technol.* **30**, 1–8 (2015). 10.1088/0268-1242/30/4/045009

[8] Kumar, M. J. & Singh, T. V. Quantum confinement effects in strained silicon MOSFETs. *Int. J. Nanosci.* **7**, 81–84 (2008). 10.1142/S0219581X08005195

[9] Khiangte, L. & Dhar, R. S. Development of tri-layered s-Si/s-SiGe/s-Si channel heterostructure-on-insulator MOSFET for enhanced drive current. *Phys. Status Solidi B* **25**, 1800034 (2018). 10.1002/pssb.201800034

[10] Khichar, S., Paptan, L., Mishra, S. & Singha, A. Strained silicon sevices: Mechanism & applications. *Int. J. Innov. Res. Comput. Commun. Eng.* **1**, 152–157 (2013).

[11] Mizuno, T. *et al.* Member IEEE electron and hole mobility enhancement in strained-Si MOSFET’s on SiGe-on-insulator substrates fabricated by SIMOX technology. *IEEE Electron. Dev. Lett.* **21**, 230–232 (2000). 10.1109/55.841305

[12] Colinge, J. P. Multi-gate SOI MOSFETs. *Microelectron. Eng.* **84**, 2071–2076 (2007). 10.1016/j.mee.2007.04.038

[13] Sharm, S. & Chaudhury, K. A novel technique for suppression of corner effect in square gate all around MOSFETs. *Electric. Electron. Eng.* **2**, 336–341 (2012).

[14] Karbalaei, M., Dideban, D. & Heidar, H. A sectorial scheme of gate-all-around field effect transistor with improved electrical characteristics. *Ain Shams Eng. J.* **12**, 755–760 (2021). 10.1016/j.asej.2020.04.015

[15] Kailasam, M. & Govindasamy, M. Impact of high-K gate dielectrics on short channel effects of DG N-Finfet. *Int. J. Sci. Technol. Res.* **9**, 2023–2026 (2020).

[16] Saeed, M. & Shahrokhabadi, M. H. Gate stack high-κ materials for Si-based MOSFETs past, present, and futures. *Microelectron. Solid State Electron.* **4**, 12–25 (2015).

[17] Goel, E. Impact of high-K gate stack on subthreshold performance of double-gate (DG) MOSFETs. *Silicon* **14**, 11539–11544 (2022). 10.1007/s12633-022-01891-5

[18] Saha, P., Dhar, R. S., Nanda, S., Kumar, K. & Alathbah, M. The optimization and analysis of a triple-fin heterostructure-on-insulator fin field-effect transistor with a stacked high-K configuration and 10 nm channel length. *Nanomaterials* **13**, 2–19 (2023). 10.3390/nano13233008 PMC10708491 38063707

[19] Karabalei, M., Daryoosh, D. & Hadi, H. Impact of high-k gate dielectric with different angles of coverage on the electrical characteristics of gate-all-around field effect transistor: A simulation study. *Results Phys.* **16**, 1–6 (2020).

[20] Bhol, K., Jena, B. & Nanda, U. Silicon nanowire GAA-MOSFET: A workhorse in nanotechnology for future semiconductor devices. *Silicon* **14**, 3163–3171 (2022). 10.1007/s12633-021-01136-x

[21] Sharma, A., Garg, N. & Kaur, G. Performance analysis of gate stacked with nitride GAA-TFET. *Mater. Today* **18**, 1683–1689 (2020).

[22] Gupta, A. *et al.* A novel approach to investigate the impact of hetero-high-K gate stack on SiGe junctionless gate-all-around (JL-GAA) MOSFET. *Silicon* **14**, 1005–1012 (2022). 10.1007/s12633-020-00860-0

[23] Goel, A., Rewari, S., Verma, S. & Gupta, R. S. High-K spacer dual-metal gate stack underlap junctionless gate all around (HK-DMGS-JGAA) MOSFET for high frequency applications. *Microsyst. Technol.* **26**, 1697–1705 (2019). 10.1007/s00542-019-04715-6

[24] Das, R. *et al.* Analysis of high-k spacer on symmetric underlap DG-MOSFET with Gate Stack architectur. *Superlattices Microstruct.* **97**, 386–396 (2016). 10.1016/j.spmi.2016.07.003

[25] Moore, M. *The International Roadmap for Devices and Systems*. 2022 edn (2022).

[26] Geumjong, B. *et al.* 3 nm GAA technology featuring multi-bridge-channel FET for low power and high performance applications. *IEEE Int. Electron. Dev. Meet.* **18437875**, 28.7.1-28.7.7 (2018).

[27] Radosavljevic, M. & Kavalieros, J. Taking Moore’s law to new heights: When transistors can’t get any smaller, the only direction is up. *IEEE Spectr.* **59**, 32–37 (2022). 10.1109/MSPEC.2022.9976473

[28] Kumar, K., Dhar, R. S., Bhattacharya, S. & Dey, R. Performance analysis and development of strain induced quantum well based nanosystem device technology. *Microsyst. Technol.* **27**, 3703–3710 (2021). 10.1007/s00542-020-05143-7

[29] Nanda, S., Dhar, R. S., Awwad, F. & Hussein, M. I. Development and analysis of a three-fin trigate Q-FinFET for a 3 nm technology node with a strained-silicon channel system. *Nanomaterials* **13**, 2–9 (2023). 10.3390/nano13101662 PMC10221103 37242078

[30] Barik, R., Dhar, R. S., Awwad, F. & Hussein, M. I. Evolution of type-II hetero-strain cylindrical-gate-all-around nanowire FET for exploration and analysis of enriched performances. *Sci. Rep.* **13**, 1–13 (2023). 37452048 10.1038/s41598-023-38239-x PMC10349044

[31] Dhar, R. S. *et al.* Direct nanoscale imaging of evolving electric field domains in quantum structures. *Sci. Rep.* **4**, 1–9 (2014). 10.1038/srep07183 PMC4246203 25431158

[32] Dhar, R. S. *et al.* Nanoscopically resolved dynamic charge-carrier distribution in operating interband cascade lasers. *Laser Photon. Rev.* **9**, 224–230 (2015). 10.1002/lpor.201400143

[33] Leys, F. E. *et al.* Epitaxy solutions for Ge MOS technology. *Elsevier* **508**, 292–296 (2005).

[34] Ritzenthaler, R. *et al.* Vertically stacked gate-all-around Si nanowire CMOS transistors with reduced vertical nanowires separation, new work function metal gate solutions, and DC/AC performance optimization. *IEEE Int. Electron. Dev. Meet. Tech. Dig.* **2**, 21.5.1-21.5.4 (2018).

[35] Drive, P. H. *Silvaco TCAD Tool Atlas Users Manual* Blg. 2, 26 Aug 2016 (2016).

